# Peripheral biomarkers of oxidative stress in aging and Alzheimer’s disease

**DOI:** 10.1590/S1980-57642009DN20100002

**Published:** 2008

**Authors:** Tania Marcourakis, Rosana Camarini, Elisa Mitiko Kawamoto, Leandro Rodrigues Scorsi, Cristoforo Scavone

**Affiliations:** 1 Department of Clinical and Toxicological Analysis, Faculty of Pharmaceutical Sciences and Neurology Investigation Center, School of Medicine (LIM-15).; 2 Department of Pharmacology, Biomedical Sciences Institute. University of São Paulo, São Paulo, Brazil.

**Keywords:** Alzheimer’s disease, aging, oxidative stress, peripheral markers, doença de Alzheimer, envelhecimento, estresse oxidativo, marcadores periféricos

## Abstract

Aging is associated with a greatly increased incidence of a number of neurodegenerative disorders, including Alzheimer’s disease (AD), Parkinson’s disease (PD) and amyotrophic lateral sclerosis (ALS). These conditions are associated with chronic inflammation, which generates oxygen reactive species, ultimately responsible for a process known as oxidative stress. It is well established that this process is the culprit of neurodegeneration, and there are also mounting evidences that it is not restricted to the central nervous system. Indeed, several studies, including some by our group, have demonstrated that increased peripheral oxidative stress markers are associated to aging and, more specifically, to AD. Therefore, it is very instigating to regard aging and AD as systemic conditions that might be determined by studying peripheral markers of oxidative stress.

## Abstract (pt)

O envelhecimento está associado a uma alta incidência de doenças neurodegenerativas, incluindo doença de Alzheimer (DA), doença de Parkinson (DP) e esclerose lateral amiotrófica (ELA). Estas condições estão relacionadas à inflamação crônica que gera espécies reativas de oxigênio, responsáveis por um processo denominado de estresse oxidativo. Está bem estabelecido que este processo está envolvido na neurodegeneração, e existem várias evidências de que ele não é restrito ao sistema nervoso central. De fato, muitos estudos, inclusive alguns de nosso grupo, demonstraram que o aumento de marcadores do estresse oxidativo periférico está associado ao envelhecimento e, mais intensamente, à DA. Assim, é muito instigante pensar no envelhecimento e na DA como doenças sistêmicas que possam ser investigadas por meio de marcadores periféricos de estresse oxidativo.

Several studies have shown that the aging process is a consequence of progressive accumulation of deleterious biochemical changes during life span, leading to an imbalance of body regulatory systems, including hormonal, immune and neuroendocrine mechanisms.^1-4^ It is believed that these changes can be more intense in neurodegenerative disorders.^5-8^

Aging is associated with a greatly increased incidence of a number of degenerative conditions, including Alzheimer’s disease (AD), Parkinson’s disease (PD), amyotrophic lateral sclerosis (ALS), atherosclerosis, and myocardial infarction. Currently, 26.6 million people worldwide have AD and this figure could rise to more than 100 million people by 2050.^9^ Most of those patients will have late-stage disease that requires a high level of care, increasing financial and personal costs with a devastating effect on the world’s economies, health-care systems and families.

More than 300 theories have been put forward to explain aging.^10^ The most accepted among these has been the “free radical theory of aging”, proposed by Denham Hartman in 1954, which states that aging is a result of the accumulation of biomolecules damaged by free radicals produced during normal metabolism. A free radical is any species capable of independent existence that contains one or more unpaired electrons, being a potent deleterious agent that leads to irreversible cellular damage and death.^11^ Nevertheless, failure of antioxidant therapy in prolonging life span in animal models suggests a much more complex phenomenon, and other factors, such as genetic background, level of physical activity and nutrition might also be affecting aging, characterizing this as a complex multi-factorial process.^12^ Nevertheless, there is agreement that free radicals can damage proteins, lipids and DNA, leading to oxidative stress, lipid peroxidation and to the formation of DNA adducts.^6,13^

Free radicals which are derivatives of oxygen (O_2_), such as superoxide anion (O_2_^•-^) and hydroxyl radical (OH^•^), are called reactive oxygen species (ROS). However, ROS, such as H_2_O_2_ are not free radicals but are also harmful. ROS are normal byproducts of the mitochondrial electron transport chain produced during respiration of aerobic organisms. When ROS are overproduced and where this production overwhelms antioxidant defense systems, cells can be damaged and this process is called oxidative stress.^14,15^

The most abundant source of ROS in the central nervous system (CNS) is the respiratory burst system of activated microglia. When the system is activated, large quantities of O_2_^•-^ are generated on the microglial external membrane, from which point they are released as a purposeful attack system. The amount of ROS produced during the process of oxidative phosphorylation represents 2% of the total oxygen consumed during respiration, but may vary depending on several parameters. The brain is particularly sensitive to oxidative damage because: 1. it is rich in the more easily peroxidizable fatty acids; 2. it is responsible for about 20% of total oxygen consumption; 3. it has high levels of iron that predispose it to Fenton reaction with the formation of OH^•^; and 4. it has high neuronal calcium input and the presence of excitatory aminoacids, such as glutamate. On the other hand, in comparison to other organs such as the liver and kidney, the brain has lower levels of the antioxidant enzymes superoxide dismutase (SOD), glutathione peroxidase (GPx) and catalase.^14,16,17^ A perfect balance among these three enzymes is necessary to maintain cell survival. Moreover, neurons also contain low levels of glutathione (GSH), a major antioxidant responsible for the elimination of cytosolic peroxides.^18^

The rapid reaction of O_2_^•-^ and nitric oxide (NO^•^) leads to the formation of peroxynitrite anion (ONOO-), and much attention has been given to this powerful oxidant agent, involved in oxidation and nitration of lipids, DNA strand leakage, nitration of proteins, including the formation of 3-nitrotyrosine and disruption of structural proteins such as actin and neurofilament L. Figure 1 shows the route of production of ONOO- as well as its antioxidant mechanism.

**Figure 1:**
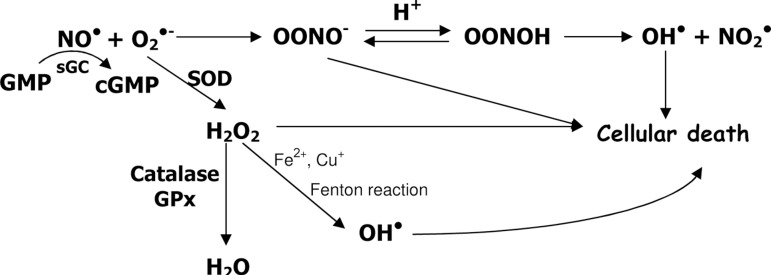
Nitric oxide (NO•) in the presence of superoxide anion (O_2_^•-^) forms peroxynitrite anion (ONOO-), which is quite instable (T_1/2_ less than 1 second) leading to the formation of hydroxyl radical (OH^•^), one of the elements responsible for cellular death. NO^•^ activates soluble guanylyl cyclase (sGC) with the formation of the second messenger cGMP. Superoxide dismutase (SOD) is responsible for the dismutation of O_2_^•-^ leading to hydrogen peroxide (H_2_O_2_) which, can be eliminated as water (H_2_O) after the action of the enzymes catalase and glutathione peroxidase (GPx). In the presence of transition metals such as iron (Fe^2+^) and copper (Cu^+^), OH^•^ can also be formed through the Fenton reaction.^19,20,21^

## Aging, neurodegeneration and oxidative stress

During the aging process, mitochondrial oxidative phosphorylation becomes less efficient, which probably contributes to an increase in the production of free radicals.^22^ The increase in O_2_^•-^ production would lead to an accumulation of intracellular calcium^23^ and activation of calcium dependent enzymes, such as nitric oxide synthase, which is responsible for the formation of NO. As a consequence of calcium accumulation, activation of protein kinase C may occur, one of the elements responsible for tau phosphorylation with the formation of the neurofibrillary tangles seen in AD^24^, or, in more extreme cases, triggers neuron apoptosis. Moreover, O_2_^•-^ formation can increase brain H_2_O_2_ production, with microglia activation leading to the formation of more free radicals and the starting of an inflammatory process.^25,26^ Most of the neurodegenerative conditions are associated with a chronic inflammation. Although there is controversy whether inflammation is causative or a consequence of the disease process, it is now clear that it can greatly influence its pathogenesis.^16,27,28^ Even though aging and neurodegeneration share the same basic mechanisms, it is difficult to establish the limits between these two processes; there are mounting evidences that neurodegeneration might be an extension of the normal aging process, which might in turn increase susceptibility to neurotoxic events.^29,30^

Each neurodegenerative process has its own neuropathological hallmarks but it has long been suspected that oxidative stress contributes to neuronal death in diseases such as AD, PD, and ALS.^15,31-34^ Moreover, it has been shown that oxidative stress is the earliest event in AD, occurring even before the development of the amyloid-β peptide (Aβ) deposit in senile plaques and accumulation of abnormal tau filaments in neurofibrillary tangles.^35-39^

The neurotoxic nature of Aβ is not well understood, however, there is evidence of the involvement of OH• and O_2_^•-^.^32,40-42^ Aβ not only can increase the levels of free radicals, but can also lead to the depletion of antioxidant agents that ultimately will determine neuronal death.^7,43^ Although the deposit of insoluble Aβ is one of the hallmarks of AD pathology, the presence of higher levels of soluble oligomers of Aβ in the brains and cerebrospinal fluid of AD patients is receiving considerable attention. Moreover, it has been shown that these soluble oligomers, which have been considered neurotoxins involved in the early pathogenesis in AD, can stimulate excessive formation of ROS through the activation of N-methyl-D-Aspartate (NMDA) glutamate receptors.^44-46^

Evidence of free radical attack in AD cortex, PD substantia nigra, and ALS spinal cord includes the presence of: 1. proteins that have been modified by glycation; 2. the existence of low molecular weight compounds that have been oxidized and nitrated (such as 3-nitrotyrosine, 3-nitro-4-hydroxyphenylacetic acid, 5-nitrotocopherol, 4-hydroxynonenal, and malondialdehyde); 3. the identification of lipids that have been peroxidated and biomarkers of DNA oxidative damage (such as 8-hydroxy-deoxyguanosine). Of those markers, 3-nitrotyrosine is quite stable and mostly derived from ONOO-, being a reliable indicator of its production.^6,33^

In order to investigate NO participation in the aging process, Özdemir et al.^47^ studied rats of different ages. They showed that brain thiobarbituric reactive substance (TBARS) production, a marker of lipid peroxidation, and nitrite levels were significantly increased with age. These increases were higher when L-NAME, a NOS inhibitor, was administered to the rats and decreased when L-arginine, a NO precursor, was given. Their conclusion was that NO had a protector effect. Whether NO will have a protector or deleterious effect, is determined by its redox status. Under physiologic conditions, NO can be present as a nitrogen monoxide (NO^•^) and/or as nitrozonium cation (NO^+^). If the cell environment is conducive for NO^•^ formation, a neurotoxic effect will be observed; however, if there is NO^+^ formation, NMDA receptor will be inhibited and a neuroprotective effect will result.^48,49^ NO behavior as a free radical or as an antioxidant agent will be dependent on O_2_•- levels. If the concentration of this anion is high, NO will lead to lipid peroxidation, otherwise NO will have an antioxidant behavior.^50-53^

## Peripheral markers of oxidative stress in aging and AD

The study of peripheral biological samples in order to identify biomarkers either in aging or in neurodegenerative disorders seems an interesting approach and several groups have been working in this field.^54^ Pratico^55^ pointed out the importance of studies on peripheral biological samples in living patients with a clinical diagnosis of AD, as it is difficult to address the question of whether oxidative stress is an early component in the pathogenesis of AD or a common final step of the neurodegenerative process based on post-mortem investigations. A follow-up of these patients in different stages of the disease could help to understand, for instance, the evolution of the antioxidant system. This kind of study received more attention in the light of the evidence that oxidative stress chronologically precedes Aβ deposit, and onset of AD symptoms. Moreover, as such changes can be detected peripherally, this points to a widespread disturbance.^36,56^

**Figure 2:**
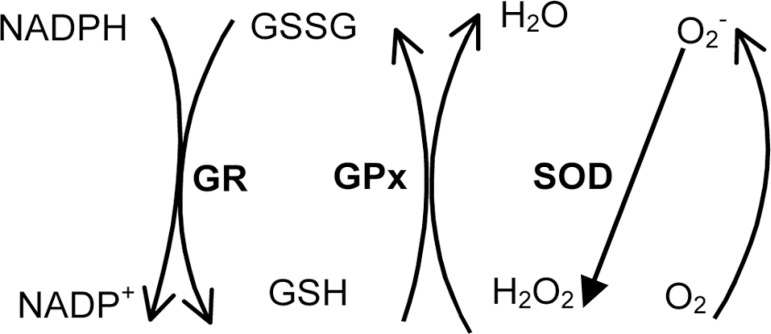
Superoxide anion (O^2^^•-^) is formed in oxidative phosphorylation in the mitochondria from molecular oxygen and is converted to H^2^O^2^ by superoxide dismutase (SOD). The detoxification of H^2^O^2^ is done by glutathione peroxidase (GPx) in the presence of reduced glutathione (GSH), leading to the formation of oxidized glutathione (GSSG). Glutathione reductase (GR) restores GSSG to GSH using NADPH.

To better understand this issue, we shall summarize some studies addressing oxidative stress in aging and AD, performed on peripheral cells. As most of the studies addressed the glutathione redox cycle, an important antioxidant system, Figure 2 can clarify the mechanisms involved.

Kasapoglu & Özben^12^ investigated 100 healthy subjects with ages ranging from 20 to 70 years and showed an age related increase in serum TBARS, a lipid peroxidation marker; however, of the erythrocytes antioxidant enzymes, only GPx had an age related reduced activity, since catalase and SOD activities were increased. Solichova et al.^57^ showed that nonagenarians have higher serum TBARS levels than subjects below 90 years’ old. Whether this oxidative state was due to a decline in antioxidant defenses or to an increased generation of ROS remains an open question.

One of the first studies to address the variability of the antioxidant enzymes SOD, GPx, and catalase during life span was performed by Guemouri et al.,^58^ in a study involving 1836 healthy subjects from 4 to 97 years of age. Below the age of 65, enzyme activities remained unchanged. However, the group detected a decrease in the activity of three enzymes, both in plasma and erythrocytes, of subjects older than 65 years of age. A similar finding was described by King et al.,^59^ who found no change in erythrocytes antioxidant enzymes of healthy volunteers with ages ranging from 35 to 69 years. Junqueira et al.^60^ evaluated 503 healthy subjects aged from 20 to more than 70 years’ old. They showed an increase in plasma TBARS levels in individuals over 50 and of erythrocyte GPx activity in those over 40 years of age. The authors reported a strong positive correlation between age and these two measures, suggesting that erythrocyte GPx could be used as a marker of oxidative stress in aging.

In a bid to understand whether healthy centenarians have a peculiar profile of non-enzymatic and enzymatic antioxidants that could explain longevity, Mecocci et al.^61^ compared the results of centenarians to subjects aged 80-99, 60-79 and below 60 years. Non-centenarians had lower levels of plasmatic non-enzymatic antioxidants (vitamins A, C and E) and increased levels of antioxidants enzymes with age. Centenarians were characterized as having the highest vitamin A and E concentrations and lower SOD activity in plasma and erythrocytes. On the basis of these results, the authors suggested that the antioxidant activity of lipid vitamins, such as A and E, is of greater relevance to longevity. Andersen et al.^62^ described a similar result as centenarians had lower SOD and higher GR activities in erythrocytes compared to 60-79 year-old controls. The centenarians with better functional capacity were the ones with higher GR activity. Paolisso et al.^63^ had the same objective and evaluated the plasma of 82 healthy subjects separated into three groups: <50 years’ old, 70 to 99 years’ old and centenarians. Centenarians had lower TBARS compared to aged subjects and showed increased GSH/GSSG ratio, vitamin C and E compared to aged subjects, which pointed to reduced oxidative stress in this group. These studies indicated that to reach extreme ages it is necessary to have a good antioxidant defense system and low oxidative stress insult.

Although not a consensus,^64^ the presence of increased oxidative stress at peripheral level in AD has been evidenced in some studies. Kawamoto et al.^65^ detected the involvement of peroxynitrite anion in platelets and erythrocyte with aging and AD. Aging was associated to an increase in TBARS and NOS activity, a decrease in basal cyclic GMP content and no change in SOD and Na,K-ATPase activities. AD patients showed a higher level of TBARS and an increase in NOS, SOD and Na,K-ATPase activities, without changes in cGMP, compared to aged controls. Na,K-ATPase is the enzyme responsible for cell response to oxidative damage, as it maintains electrochemical sodium and potassium gradient. Its catalytic a subunit is sensitive to damage by free radicals while ONOO- has been reported to cause cell membrane damage, which in turn leads to the disruption of Na,K-ATPase activity.^66-69^ The results presented by Kawamoto et al.^65^ demonstrated a disruption in systemic modulation of oxidative stress in aging and at greater intensity in AD, and are consistent with the suggestion that neurodegeneration and aging could share a common pathogenic pathway.

Praticó et al.^70^ described an increase in isoprostane generation, an index of lipid peroxidation, in urine, blood and cerebrospinal fluid (CSF) of AD patients. Besides, urinary and blood isoprostane correlated with CSF levels in AD patients. In fact, some other groups have also demonstrated an increase in peripheral lipid peroxidation index, such as TBARS, malondialdehyde or isoprostanes in AD.^71-74^

Oxidative DNA damage, as a measure of oxidative stress, has also been the subject of investigation. Meccocci et al.^75^ showed an increase in lymphocyte DNA damage in AD patients compared to healthy controls. This same evidence was corroborated by Migliore et al.,^36^ who described not only an increase in the amount of oxidized DNA bases in peripheral leukocytes of AD, but also in mild cognitive impairment (MCI) subjects, versus age-matched controls. The fact that this damage was detected in individuals with MCI provides a new finding in the direction of the hypothesis that oxidative stress can be present from the beginning of neurodegeneration. Furthermore, Rinaldi et al.^76^ described decreased levels of non-enzymatic (vitamin A, C and E; α and β-carotene) in plasma and enzymatic antioxidants (SOD and GPx) in erythrocytes of AD and MCI patients, lending further weight to the findings that MCI is a prodromal stage of AD. In a recent study, Gackowski et al.^77^ showed a similar result regarding oxidative DNA damage in mixed AD and vascular dementia (AD/VD), suggesting a common route in the pathogenesis of AD and AD/VD.

With the aim of finding a relationship between cognitive function and lipid peroxidation in erythrocytes, Delibas et al.^73^ examined a group of AD patients at an interval of five years. They found a negative correlation between MDA and the scores on the Mini Mental State Examination (MMSE), suggesting that lipid peroxidation might be one of the factors responsible for cognitive deterioration.

Zafrilla et al.^74^ analyzed oxidative stress at different stages of AD. Lipid peroxidation was higher in patients at the advanced stage of illness than in controls; however, no difference between light/moderate and advanced AD was detected. The total antioxidant plasmatic status of AD patients at any stage was lower than in the control group. Also, at different stages of the disease, GPx activity was increased and GR decreased.

In conclusion, although several studies support the presence of peripheral oxidative damage in AD, it is not clear if neurodegeneration is a process in which peripheral oxidative stress is an active participant or a simple bystander.

Despite mounting evidence of oxidative stress involvement in AD, antioxidant therapies remain quite controversial. However, no effect in preventing AD was reported by several groups after healthy subjects had received antioxidants supplementation.^78-80^ Moreover, Cole et al.^81^ found no effect with nonsteroidal anti-inflammatory drugs and vitamin E supplement in controlling oxidative damage. Finally, the meta-analysis performed by Miller et al.^82^ showed that high doses of vitamin E were associated with increased mortality. Unfortunately, a good theory supported by strong basic scientific data is not enough to guarantee effective therapy.
